# Genetic variants associated with idiopathic Parkinson’s disease in Latin America: A systematic review

**DOI:** 10.1007/s10048-025-00817-8

**Published:** 2025-04-03

**Authors:** Felipe Duarte-Zambrano, David Felipe Alfonso-Cedeño, Jorge A. Barrero, Luis Alejandro Rodríguez-Vanegas, Valentina Moreno-Cárdenas, Anamaría Olarte-Díaz, Gonzalo Arboleda, Humberto Arboleda

**Affiliations:** 1 Semillero de Investigación en Neurociencias, Facultad de Medicina, Universidad Nacional de Colombia, Bogotá, Colombia; 2 Grupo de Investigación en Neurociencias y Muerte Celular, Facultad de Medicina E Instituto de Genética, Universidad Nacional de Colombia, Bogotá, Colombia; 3 Departamento de Patología, Facultad de Medicina, Universidad Nacional de Colombia, Bogotá, Colombia; 4 Departamento de Pediatría E Instituto de Genética, Facultad de Medicina, Universidad Nacional de Colombia, Bogotá, Colombia

**Keywords:** Systematic review, Genetic association studies, Parkinson’s disease, Latin America, Genetic architecture, Complex disease, Candidate gene, GWAS, XWAS

## Abstract

Idiopathic Parkinson’s disease (PD) constitutes a complex trait influenced by genetic, environmental, and lifestyle factors, with an estimated heritability of nearly 30%. However, a large proportion of the heritable variation linked to PD remains uncertain, partly due to ancestral bias. Expanding research into Hispanic populations can contribute to address this gap. To review the evidence of genetic variants associated with idiopathic PD in Latin America. A PRISMA-compliant systematic review was conducted in MEDLINE, EMBASE and LILACS, compiling studies published up to February 7, 2025. Nineteen case–control studies were included. Two hypothesis-free studies identified rs525496 near *H2BW1* as a protective factor and rs356182 in *SNCA* as a risk factor through XWAS and GWAS, respectively. Seventeen hypothesis-driven studies examined over three hundred variants, identifying nineteen genetic markers; risk factors included one INDEL in *NR4A2*, CNV burdens in *PRKN*, *SNCA*, and *PLA2G6*, along with fourteen variants in six loci including *GBA*, *APOEε4*, *MTHFR*, *LRRK2*, and *SNCA*. Three SNPs in the *PICALM*, *ALDH1A1*, and *APOE-ε3* loci were identified as protective factors. Additionally, six *SNCA* variant haplotypes appear to increase PD risk, while two *NR4A2* INDELs haplotypes showed mixed effects. This review summarized genetic loci associated with idiopathic PD in Latin American populations evidencing an overlap with European findings as well as novel loci, although awaiting replication and validation. These observations contribute to the understanding of genetic configuration of the disease and highlight the need for further genomic research in underrepresented groups that include local ancestry analysis within admixed cohorts to guide development of personalized treatments and population-specific interventions.

**Supplementary Information:**

The online version contains supplementary material available at 10.1007/s10048-025-00817-8.

## Introduction

The complex phenotypic expression of idiopathic Parkinson’s disease (PD) is underpinned by the intricate interactions between genes, environment, and lifestyle factors. From a disease genetics perspective, it is essential to comprehend both the deterministic mutations for familial PD, and the genetic risk factors for idiopathic PD expression. As for familial PD, the identification of monogenic causal mutations such as SNCA, PINK1, DJ1, VPS35, VPS13C has aided in the understanding of the disease pathophysiology and identification of potential therapeutic targets; yet, these account for merely 10% of PD cases [1–3]. On the other hand, multiple genetic variants have been shown to modify the risk for idiopathic PD, the most common version of PD cases, with an estimated heritability within 16–36% depending on the disease prevalence of each population [4]. However, more than half of this polygenic variance remains unaccounted for in current genome-wide association studies [5]. As such, approximately 90 genetic susceptibility loci have been identified in European population [4], along with 78 risk loci in the most recent multi-ancestry genome-wide association meta-analysis [6], from which *SNCA, LRRK2* and *GBA* stand out as risk factors for idiopathic PD [7].

Since the advent of genome-wide association studies (GWAS) in the early 2000s, the field of PD genomics has gained extensive insight into the genetic architecture of idiopathic PD; nonetheless, most of the variants that comprise the expected heritability observed in GWAS [4] and longitudinal twin studies [8] have not been identified. The missing heritability of PD may arise from multiple factors; omission of gene epistasis and molecular interactions [9, 10]; contribution of rare variants with high effect sizes in the “common disease-rare variant” hypothesis in contrast to the classical view of “common disease-common variant hypothesis” used in GWAS [5]; the need of greater cohorts to identify small effect sizes of common variants due to loss of power when correcting for multiple comparisons [11]; structural variants and complex genomic regions difficult to assess with current genotyping and short read sequencing approaches [12] and ancestral bias of current genomic research centered in European populations [7]. Particularly, the latter excludes underrepresented populations which could account for a significant contribution to the missing heritability of PD [13]. As understudying local ancestry within admixed cohorts underpowers genome-wide variance analysis [14]. Moreover, due to different linkage disequilibrium traits within distinct ancestries, specific variants could be missed, resulting in an incomplete disease genetic architecture [15].

In this sense, findings from European studies are hardly generalizable to all affected subjects, the pathophysiological inferences can be disproportionate, and the application of new diagnostic and therapeutic strategies based on genetic profiles is limited [13]. Hence, it is essential to extend genomic studies to underrepresented populations, to complement and enrich the genomic architecture of the disease [16]. Consequently, multiple national and international investigations have been carried out in the Latin American region, which have shown discordant and complementary results to the outcomes in populations that have been classically studied [16]. However, to date there is no compilation of evidence on the genetic variants associated with PD in the Latin American population. Therefore, assessing the overall findings of Hispanic genetic association studies allows for the unification of these efforts, identifying the status and direction needed for genomic research in Latin America. The present study aimed to compile the genetic variants associated with idiopathic PD in Latin America through a systematic review of genetic association studies to contribute to the identification of variants with a risk or protective association with idiopathic PD.

## Methodology

A systematic review of genetic association studies was conducted and reported based on the Preferred Reporting Items for Systematic Reviews (PRISMA) parameters [17].

### Eligibility criteria

Original studies written in English and Spanish, with no publication time restriction, were considered eligible for inclusion to compile the largest number of reported genetic variants associated with idiopathic PD phenotype in the Latin American population. Analytical observational and genetic association studies were included to evaluate the distribution of genotypes and/or allele frequencies of genetic variants, as well as the risk or protection of developing idiopathic PD in relation to the presence of the variants based on association measures such as odds ratio (OR) or risk ratio (RR). Different types of genetic variants were considered: single nucleotide polymorphisms (SNPs), copy number variations (CNVs), variable number tandem repeats (VNRTs), insertion and deletion mutations (INDELs). Furthermore, studies were excluded based on the following criteria: literature review studies, case reports or series, postmortem investigations or in non-Latino populations, and studies in subjects with explicit family history of definite PD or with monogenic variants of the disease, secondary parkinsonian syndromes, or with inadequate diagnostic definition of idiopathic PD.

### Search strategy and information sources

The systematic literature search considered articles published up to February 7, 2025, and was performed in the MEDLINE database through PubMed, EMBASE through the Elsevier platform, and LILACS through the *Biblioteca Virtual en Salud* (BVS) platform. Search strategies constructed from MeSH, Emtree, and DeCS terms with their respective synonyms and free terms joined by Boolean operators (“AND” and “OR”) were employed as presented in the following algorithm: ("Latin American countries") AND (("gene locus") OR ("genotype") OR ("mutation") OR ("genetic variation") OR ("genetic variability") OR ("genetic polymorphism") OR ("single nucleotide polymorphism") OR ("indel mutation") OR ("copy number variation") OR ("variable number of tandem repeat") OR ("haplotype") OR ("haplogroup") OR ("gene linkage disequilibrium")) AND (("Parkinson Disease") OR ("Idiopathic Parkinson’s Disease") OR ("Lewy Body Parkinson’s Disease") OR ("Parkinson’s Disease, Idiopathic") OR ("Parkinson’s Disease, Lewy Body") OR ("Parkinson Disease, Idiopathic") OR ("Parkinson’s Disease") OR ("Idiopathic Parkinson Disease") OR ("Lewy Body Parkinson Disease") OR ("Primary Parkinsonism") OR ("Paralysis Agitans")). Detailed search strategies are presented in APPENDIX 1.

### Selection process

Assessment of records independently by more than one reviewer was conducted for the study selection process. The systematic search in the databases consulted was performed by three authors (FDZ, DFAC and JAB). Titles and abstracts of the retrieved records were compiled in the Mendeley reference manager program, and the management of records including duplicates elimination was carried out in the Rayyan® software. Subsequently, nine reviewers (FDZ, DFAC, JAB, LARV, VMC, AOD) independently screened all titles and abstracts to exclude articles with no relevance to the review objective. Studies selected by consensus based on title and abstract were further assessed independently by the same reviewers based on full text evaluation to determine their final inclusion. Disagreements in the study selection process were resolved by consensus and, when necessary, an additional reviewer (HA) was consulted. The reasons for exclusion of studies during the full-text assessment are presented in APPENDIX 2.

### Data items and data collection process

The following data were extracted for each record:

- Author(s) and year of publication.
- Type of study.
- Latin American country.
- Population characteristics.
- Diagnostic criteria for idiopathic PD.
- Genetic variants studied.
- Association of the variants with the development of idiopathic PD.

### Risk of bias assessment

The risk of bias of the included studies was evaluated by three reviewers (FDZ, LARV and VMC) using the Quality of Genetic Studies (Q-Genie) tool [18]. This assessment tool was used to establish the methodological quality of genetic association studies based on the classification according to scores as follows: (a) studies with a control group: low quality (≤ 35 points), moderate quality (36–45 points), and high quality (> 45 points), (b) studies without a control group: low quality (≤ 32 points), moderate quality (33–40), and high quality (> 40 points).

### Effect measures and data synthesis

The outcome of idiopathic PD risk was analyzed using measures of association including odds ratio (OR) or relative risk (RR) as the primary outcome estimator. Included studies were discussed separately according to geographical regions across Latin America, specific genetic loci that contained different types of mutations of interest and the type of methodological study, in the case of hypothesis free studies (e.g., GWAS) special focus was directed towards the replicated results within the studies.

## Results

**Fig. 1:**
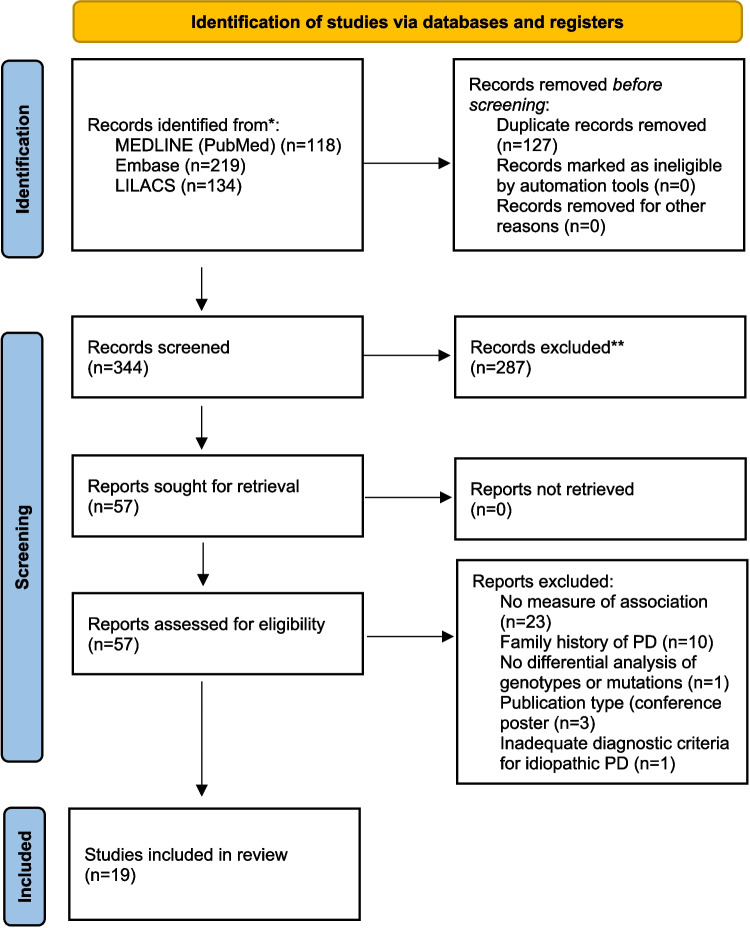
PRISMA flow diagram of study selection process

A total of 471 studies were identified in the initial database search. Following the elimination of 127 duplicate records, 344 studies were evaluated by title and abstract. Of these, 57 articles were selected for full-text evaluation. Finally, 19 studies were included in the review. Figure 1 presents the study selection process. The list of excluded records and the reason for exclusion is presented in Appendixs 2 and 3.

**Fig. 2:**
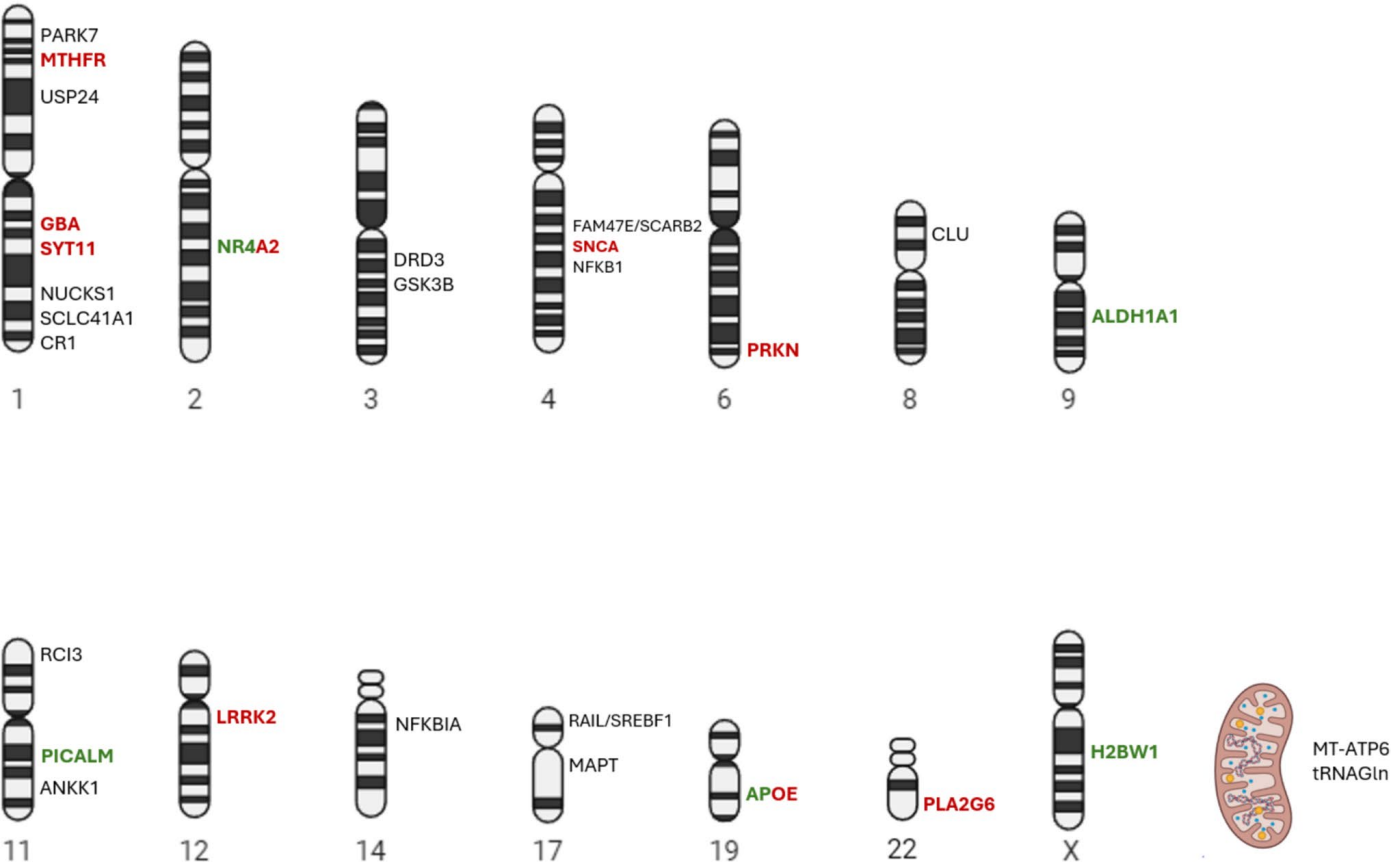
Visual display of studied loci in Latino population with idiopathic PD in genetic association studies. Green: protective association; Red: risk association; Grey: loci not significantly associated with PD risk. Source: own elaboration, BioRender

The characteristics of the studies included in this review are synthesized in Table 1 presenting the main characteristics of the population, diagnostic criteria for idiopathic PD, the studied mutations and their association status with PD of each included study. Likewise, a schematic summary of the main genetic loci identified is depicted in Fig. 2, color-coded with their risk association status with PD. Genetic variants associated with idiopathic PD sorted by Latin American region are summarized in Table 2, and candidate gene loci with non-statistically significant associations are presented in Appendix 4. Moreover, after the risk of bias assessment and based on the score obtained, 10 studies were classified as high quality, 8 studies as moderate quality, and 1 study as low quality.

**Table 1:** Characteristics of included studies

Author	Region	Population	Diagnostic criteria	Genetic Variants	Association status with PD	Type of study	Quality
Santos-Reboucas et al. (2017) [19]	Brazil	PD group (n = 166): 103 men and 63 women (69.0 ± 8.4 years) Control group (n = 176): 47 men and 129 women (70.7 ± 6.04 years)	UK Parkinson’s Disease Society Brain Bank Diagnostic Criteria (UKPDSBB)	SNPs: ***PICALM:*** rs3851179: chr11.hg38:g.86157598G > A ***CR1:*** rs3818361: chr1.hg38:g.207611623G > A ***CLU:*** rs11136000: chr8.hg38:g.27607002C > T	***PICALM* rs3851179:** AA genotype: OR: 0.34 (95% CI: 0.14–0.81) (p = 0.01) A allele: OR 0.67 (95% CI: 0.48–0.94) (p = 0.02) *CR1* & *CLU* loci were NOT significantly associated with PD risk*	Case – Control study (hypothesis driven)	High
Leal et al. (2023) [20]	Brazil, Chile, Colombia, Perú and Uruguay (LARGE-PD consortium)	**Discovery cohort** (LARGE-PD cohort) (n = 1498): PD group (n = 798): 424 men and 374 women (59.3 ± 13.9 years) Control group (n = 683): 230 men and 453 women (59.3 ± 13.9 years) **Replication cohort** (IPDGC + Bambuí) (ethnically matched) (n = 949) PD cases (n = 127): 79 men and 48 women Control group (n = 822): 291 men and 531 women	UK Parkinson’s Disease Society Brain Bank Diagnostic Criteria (UKPDSBB)	**X-chromosome-wide association study**	***H2BW1* rs525496: chrX.hg38:g.103929276–104031236C > T:** T allele (Discovery cohort): OR: 0.6 (95% CI:0.478–0.77) (p = 3.13 × 10–5) T allele (replication cohort): OR: 0.6 (95% CI:0.37–0.98) (p = 0.04)	Case – Control (hypothesis free)	High
Sarihan et al. (2021) [21]	Brazil, Chile, Colombia, Perú and Uruguay (LARGE-PD consortia)	PD group (n = 747): 395 men and 252 women (62 years); Age at onset 54.4 years Grupo control (n = 632): 202 men and 423 women (56.6 years)	UK Parkinson’s Disease Society Brain Bank Diagnostic Criteria (UKPDSBB)	**4 classes of autosome CNV burden** Overall CNV burden (including CNVs in nongenic regions); CNVs burden intersecting any gene except PD genes; CNVs burden intersecting 19 known PD-related genes: *LRRK2, PRKN, PARK7, PINK1, SNCA, VPS35, GBA, ATP13A2, DNAJC6, DNAJC13, EIF4G1, FBXO7, GIGYF2, HTRA2, PLA2G6, RAB39B, SYNJ1, TMEM230 and VPS13C* Large CNVs (> 1 Mb) burden	**CNVs burden intersecting PD-related genes (mainly driven by CNVs in PRKN, SNCA and PLA2G6):** OR: 3.97 (95% CI: 1.69–10.5) (p = 0.018) Overall CNV burden: not significantly associated with PD risk* CNVs burden intersecting any gene except PD genes: OR: not significantly associated with PD risk* Large CNVs burden: OR: not significantly associated with PD risk*	Case – Control study (hypothesis free & driven)	High
Salas-Leal et al., (2019) [22]	México	PD group (n = 120): 61 men and 59 women (70.5 ± 9.35 years); Age at onset of PD: 65.29 ± 9.22 years Control group (n = 178): 90 men and 88 Women, (69.26 ± 8.95 years)	UK Parkinson’s Disease Society Brain Bank Diagnostic Criteria (UKPDSBB)	SNP ***ALDH1A1*** rs3764435: chr9.hg38:g.72901960A > C	***ALDH1A1* rs3764435:** AA vs CC genotype: adjusted OR: 0.40 (95% CI:0.19–0.84) (p = 0.016) AA + AC vs CC genotypes: adjusted OR:0.04 (95% CI: 0.21–0.75) (p = 0.005) Adjusted ORs: for age, sex, smoking, alcoholism, metal and pesticide exposure, well-water, consumption and recruitment site	Case – Control study (hypothesis driven)	Moderate
Romero-Gutiérrez et al. (2021) [23]	México	PD group (n = 118): 60 men and 58 women (69.92 ± 10.01 years); Age at onset of PD: 64.08 ± 10.46 Control group (n = 193): 97 men and 96 women (69.80 ± 8.63 years)	UK Parkinson’s Disease Society Brain Bank Diagnostic Criteria (UKPDSBB)	SNPs ***USP24*** rs13312: chr1.hg38:g.55067069G > C ***PARK7*** rs3766606: chr1.hg38:g.8022197G > T ***PRKN*** rs1801474: chr6.hg38:g.162201165C > T& rs1801582: chr6.hg38:g.161386823C > G ***ANKK1*** rs1800497: chr11.hg38:g.113400106C > T ***MTHFR*** rs1801133: chr1.hg38:g.11796321C > T ***GSK3B*** rs334558: chr3.hg38:g.120094435A > G ***DRD3*** rs6280: chr3.hg38:g.114171968C > T ***MAPT*** rs242562: chr7.hg38:g.45949373A > G ***SNCA*** rs2736990: chr4.hg38:g.89757390C > T, rs356220: chr4.hg38:g.89720189C > T & rs356219: chr4.hg38:g.89716450A > G ***LRRK2*** rs1491942: chr12.hg38:g.40227006C > G, rs33949390: chr12.hg38:g.40320043C > G & rs34778348: chr12.hg38:g.40363526A > G ***FAM47E***: rs6812193: chr4.hg38:g.76277833C > T ***SREBF1*** rs11868035: chr17.hg38:g.17811787C > T	***LRRK2* rs1491942:** Additive model of genotypes: adjusted OR: 1.71 (95% CI: 1.22–2.40) (p = 0.002) ***MTHFR* rs1801133:** Additive model of genotypes: adjusted OR 1.54 (95% CI: 1.11–2.15) (p = 0.01) *USP24, PARK7, ANKK1, GSK3B, DRD3, MAPT, SNCA, FAM47E, SREBF1* loci were NOT significantly associated with PD risk* Adjusted ORs: for age, sex and ancestry	Case – Control study (hypothesis driven)	High
García et al. (2019) [24]	México	PD group (n = 175): 100 men and 75 women (62.97 ± 10.96 years) Control group (n = 194): 71 men and 123 women (68.48 ± 6.04 years)	Queen Square Brain Bank criteria	SNPs tRNAGln: m.4336 T > C ***MT-ATP6:*** m.8701G > A	tRNAGln and *MT-ATP6* loci were NOT significantly associated with PD risk*	Case – Control study (hypothesis driven)	Low
Ramírez—Jiranoa et al. (2007) [25]	México	PD group (n = 51) (61.3 years (range 43–86 years)) Control Group (n = 121) (49.2 years (range 21–86 years))	Part III of the Unified Scale for Parkinson’s Disease	SNP SNCA: IVS4 + 66A-G (rs-ID not available)	SNCA loci was NOT significantly associated with PD risk*	Case – Control study (hypothesis driven)	Moderate
Salas-Leal et al. 2021 [26]	Mexico	PD Group (n = 88): 42 women and 46 men (70.14 ± 9.29 years); Age at onset: 65.07 ± 9.6 years) Control Group (n = 88): 42 women and 46 men (70.47 ± 9.41 years)	UK Parkinson’s Disease Society Brain Bank Diagnostic Criteria (UKPDSBB)	SNP ***SNCA***: rs356219: chr4.hg38:g.89716450A > G	**SNCA rs356219:** G allele: adjusted OR: 1.80 (95% CI:1.14–2.83) (p = 0.011) G/G genotype: adjusted OR: 2.87 (95% CI:1.14–7.25) (p = 0.025) A/A + A/G genotype: adjusted OR: 2.49 (CI:1.29–4.8) (p = 0.006) Adjusted ORs: for sex and age	Case – Control study (hypothesis driven)	Moderate
Gallegos-Arreola et al. 2009 [27]	Mexico	PD group (n = 105): 63 men and 42 women (63 ± 9 years) Control group (n = 107): 47 men and 60 women (50 ± 14 years)	Subjects with at least two of the four cardinal signs: (1) resting tremor, (2) bradykinesia, (3) rigidity, (4) postural instability. And no other apparent cause of Parkinsonism	SNPs ***APOEε2:*** rs7412: chr19.hg38:g.44908822C > T ***APOEε4:*** rs429358: chr19.hg38:g.44908684C > T ***APOEε3:*** (wild type)	***APOEε3* & *APOEε4*:** ε3/ ε3 Genotype: adjusted OR: 0.345 (95% CI 0.153–0.776) (p = 0.010) ε3/ε4 Genotype: adjusted OR: 2.917 (95% CI 1.236–6.881) (p = 0.015) ε3 Alle: OR 0.36 (95% CI 0.20–0.61) (p = 0.001) ε4 Alle: OR 2.57 (95% CI 1.42–4.79) (p = 0.001) Adjusted ORs: for age, smoking, caffeine consumption, exposure to herbicides, pesticides and solvents	Case – Control study (hypothesis driven)	Moderate
Marca et al. 2013 [28]	Perú	PD group (n = 163): 81 men and 82 women (61.35 ± 10.19 years) Age of onset: (56.23 ± 10.47 years) Control group (n = 176): 79 men and 97 women (54.61 ± 14 years)	UK Parkinson’s Disease Society Brain Bank Diagnostic Criteria (UKPDSBB)	SNP ***APOEε4:*** rs429358: chr19.hg38:g.44908684C > T	*APOE ε4* was NOT significantly associated with PD risk*	Case – Control study (hypothesis driven)	Moderate
Baltus et al. 2021 [29]	Brazil	PD group (n = 55): 23 men and 32 women (69.51 ± 10.46 years) Control group (n = 55): 25 men and 30 women (71.85 ± 9.99 years)	UK Parkinson’s Disease Society Brain Bank Diagnostic Criteria (UKPDSBB)	SNP ***NFKBIA:*** rs696: chr4.hg38:g.35401887C > G INDEL ***NFKB1:*** rs28362491: chr4.hg38:g.102500998-94ATTG	*NFKB1* loci was NOT significantly associated with PD risk* *NFKBIA* loci was NOT significantly associated with PD risk*	Case – Control study (hypothesis driven)	High
Ruiz-Sánchez et al. 2017 [30]	Mexico	PD group (n = 227): 123 men and 104 women (62.01 ± 10.6 years) Age at onset: 55.48 ± 10.37 years Control group (n = 454): 246 men and 208 women (62.63 ± 11.2 years)	UK Parkinson’s Disease Society Brain Bank Diagnostic Criteria (UKPDSBB)	INDELs ***NR4A2***: rs35479735: chr12.hg38:g.156326700–156326702 & rs34884856: chr2.hg38:g.156333105–156333106 ***NR4A2* haplotypes:** with rs34884856 + rs35479735	***NR4A2* rs35479735 (2G/3G):** 3G/3G genotype: adjusted OR: 1.53 (95% CI: 1.08–2.19) (p = 0.018) ***NR4A2* rs34884856 (2C/3C) + rs35479735 (2G/3G) haplotypes:** (3C + 2G): OR: 0.78 (95% CI 0.62–0.98) (p = 0.037) (2C + 3G): OR: 1.28 (95% CI: 1.02–1.60) (p = 0.030) rs34884856 alone was NOT significantly associated with PD risk* Adjusted OR: for sax and age	Case – control study (hypothesis driven)	Moderate
García et al. 2015 [31]	Mexico	PD group (n = 140): 95 men and 45 women (65.46 ± 11.5 years) Age at onset: 55.48 ± 10.37 years Control group (n = 216): 140 men and 76 women (63.68 ± 8.8 years)	Queen Square Brain Bank criteria	SNP ***MTHFR***: rs1801133: chr1.hg38:g.11796321C > T	***MTHFR* rs1801133:** CC genotype: adjusted OR: 2.06 (95% CI 1.101–3.873) (p = 0.024) Adjusted OR: for smoking and gender	Case – control study (hypothesis driven)	Moderate
Loesch et al. 2021 [32]	Brazil, Chile, Colombia, Perú and Uruguay (LARGE-PD consortium)	Discovery cohort (LARGE-PD cohort): (n = 1497) PD group (n = 807): 428 men and 379 women (61.70 years (SD: 12.81 years)), Age at onset: 54.09 years (SD: 14.35) Control group (n = 690): 228 men and 462 women (56.48 years (SD: 14.59 years)) Replication cohort (23andMe): (n = 440.756) PD group (n = 1234) Control group (n = 439.522)	UK Parkinson’s Disease Society Brain Bank Diagnostic Criteria (UKPDSBB)	**Genome wide association study**	**SNCA rs356182: chr4.hg38:g.89704960C > T:** G (Discovery): OR: 1.58 (CI:1.35–1.86) (p = 2.48 × 10–8) G (replication): OR: 1.26 (CI:1.16–1.37) (p = 4.55 × 10–8)	Case – control study (hypothesis free)	High
Sesar et al. 2016 [33]	Mexico	PD group (n = 271) Control group (n = 260)	UK Parkinson’s Disease Society Brain Bank Diagnostic Criteria (UKPDSBB)	SNPs **SYT11**: rs822508: chr1.hg38:g.155850558 T > C, rs729022: chr1.hg38:g.155882332C > T, rs34372695: chr1.hg38:g.156060246C > T & rs12563627: chr1.hg38:g.155862966 T > C	***SYT11* rs822508** C allele: OR: 1.33 (95% CI: 1.05–1.68) (p = 0.017) ***SYT11* rs729022** A allele: OR: 1.29 (95% CI 1.02- 1.64) (p = 0.032) ***SYT11* rs34372695** T allele: OR: 2.07 (95% CI 1.24–3.87) (p = 0.00003) rs12563627 loci was NOT significantly associated with PD risk*	Case – Control study (hypothesis driven)	High
Velez-Pardo et al. 2019 [34]	Colombia and Peru	Colombian PD group (n = 131) 63 males (64.6 ± 13.4 years); Age at onset: 49.3 ± 16.4 years Colombian control group (n = 164) 82 males (53.8 ± 14.1 years) Peruvian PD group (n = 471) 258 males (62.1 ± 12.2 years) Age at onset: 57.1 ± 13.2 Peruvian control group (n = 155) 49 males (54 ± 12.8 years) Combined cohort PD group (n = 602) 321 males (62.6 ± 12.5 years) Age at onset: 55.4 ± 14.3 Combined cohort control group (n = 319) 131 males (53.9 ± 13.5 years)	UK Parkinson’s Disease Society Brain Bank Diagnostic Criteria (UKPDSBB)	SNPs ***GBA***: rs773409311: chr1.hg38:g.155238186 T > C, rs421016: chr1.hg38:g.155235252A > G, rs76763715: chr1.hg38:g.155235843C > T, rs2230288: chr1.hg38:g.155236376A > G, rs398123530: chr1.hg38:g.155238597G > A, rs439898: chr1.hg38:g.155238630C > T & p.R47X (rs-ID not available)	**GBA rs773409311** C allele in Colombian cohort: adjusted OR: 6.5 (CI 1.2–36.3) Rest of GBA loci were NOT significantly associated with PD risk*, in both Colombian and Peruvian cohorts Adjusted OR: for age and sex	Case – Control study (hypothesis driven)	Moderate
Dávila-Ortiz de Montellano et al. 2016 [35]	Mexico	PD group (n = 171) 111 men and 60 women (51.6 ± 13.4 years) Control group (n = 171)	UK Parkinson’s Disease Society Brain Bank Diagnostic Criteria (UKPDSBB)	SNPs ***SNCA***: rs356220: chr4.hg38:g.89720189C > T, rs356203: chr4.hg38:g.89744890C > T, rs7684318: chr4.hg38:g.89733852 T > C, rs2736990: chr4.hg38:g.89757390A > G, rs2619364: chr4.hg38:g.89838736A > G, rs2619363: chr4.hg38:g.89837896G > T, rs17016074: chr4.hg38:g.89726127G > A & rs356219: chr4.hg38:g.89716450A > G ***SNCA* haplotypes** with rs356220 + rs356203 + rs7684318 + rs2736990	***SNCA* rs356220** T allele: OR: 2.1 (95% CI: 1.35–3.27) (p = 0.0024) ***SNCA* rs356203** T allele: OR: 1.6 (95%CI: 1.03–2.47) (p = 0.035) ***SNCA* rs7684318** T allele: OR: 9.94 (95% CI 5.99–16.5) (p < 0.001) ***SNCA* rs2736990** G allele: OR: 2.42 (95% CI 1.55–3.76) (p < 0.001) ***SNCA* haplotypes rs356220 + rs356203 + rs7684318 + rs2736990:** C + G + T + C: OR: 1.92 (95% CI:1.18–3.13) (p = 0.009) C + A + T + C: OR: 2.54 (95% CI 1.37–4.72) (p = 0.003) C + G + C + C: OR: 14.51 (95% CI 1.68–125.15) (p = 0.016) T + G + T + C: OR: 8.97 (95% CI 2.05–39.19) (p = 0.003) T + A + C + C: OR: 4.71 (95% CI 1.16–19.15) (p = 0.031) C + A + C + T: OR: 16.18 (95% CI 1.88–139.18) (p = 0.012) Rest of SNCA loci were NOT significantly associated with PD risk*	Case – Control study (hypothesis driven)	Moderate
Garcia et al. 2016 [36]	Mexico	PD group (n = 106) 75 men and 31 women (62.52 ± 12.1y); Age at onset: 56.26 ± 14.4 years Control group (n = 135) 89 men and 46 women (65.73 ± 9.4 years)	Queen Square Brain Bank criteria	SNP *A***SNCA: **rs3857059: chr4.hg38:g.89754087A > G	***SNCA* rs3857059:** GG genotype: OR: 2.4 (95% CI 1.121–5.138) (p = 0.02)	Case – control study (hypothesis driven)	Moderate
Brolin et al. 2022 [37]	LARGEPD population	PD group (n = 798) 424 men and 424 women (61.7 (12.8) years); age at onset: 54.1 (14.4) years Control group (n = 683) 230 men and 453 women (56.5 (14.6) years)	UK Parkinson’s Disease Society Brain Bank Diagnostic Criteria (UKPDSBB)	SNPs 293 polymorphisms in *RIC3*	*RIC3* loci was NOT significantly associated with PD risk*	Case – control study (hypothesis driven)	High

2C: deletion C; 3C: insertion C;2G:deletion G; 3G: insertion G.*ALDH1A1*: Aldehyde Dehydrogenase 1; *ANKK1*:ankyrin repeat and kinase domain containing 1; *APOE*: Apolipoprotein E; *ATP13A2*:ATPase cation transporting 13A2; *CLU*: Clusterin; *CR1*: Complement Receptor 1; *DRD3*: Dopamine receptor D3; *DNAJC6*: DnaJ heat shock protein family (Hsp40) member C6; DNAJC13: DnaJ (Hsp40) homolog, subfamily C, member 13; *EIF4G1*: Eukaryotic translation initiation factor 4 gamma 1; *FAM47E*: family with sequence similarity 47 member E; *FBXO7*: F-box only protein 7; *GBA*: glucocerebrosidase; *GIGYF2*: GRB10 Interacting GYF Protein 2; *GSK3B*: Glycogen synthase kinase-3 beta; *H2BW1*: H2B Histone Family Member W; *HTRA2*: HtrA serine peptidase 2; IPDGC;LARGE-PD; Latin America Research Consortium on the Genetics of Parkinson’s Disease; *LRRK2*: Leucine-rich repeat kinase 2; *MAPT*: microtubule associated protein tau; MT-ATP6: mitochondrially encoded ATP synthase membrane subunit 6; *MTHFR*: methylenetetrahydrofolate reductase; NR4A2: nuclear receptor subfamily 4 group A member 2; NFKB1: nuclear factor kappa B subunit 1; NFKBIA: NFKB Inhibitor Alpha; *PARK7*: Parkinsonism associated deglycase (DJ-1 gene);* PICALM*: Phosphatidylinositol binding clatrhin assembly protein; *PLA2G6*: phospholipase A2 group VI; *PINK1*: PTEN induced putative kinase 1; *PRKN*: Parkin (PARK2 gene); *RAB39B*: RAB39B, member RAS oncogene family; RIC3: acetylcholine receptor chaperone; *SNCA*: alpha-synuclein; *SREBF1*: Sterol regulatory element-binding protein 1; *SYNJ1*: synaptojanin 1; *SYT11*: synaptotagmin 11; tRNAGln: mithocondrial transfer RNA for the Gln amino acid; *TMEM230*: transmembrane protein 230 *USP24*: ubiquitin specific peptidase 24; *VPS35*: Vacuolar protein sorting ortholog 35; *VPS13C*: vacuolar protein sorting 13 homolog C; *Odds ratio value non statistically significant.

**Table 2:** Summary of genetic variants associated with idiopathic PD sorted by Latin American region

Region	Loci
Colombia	**SNPs** *GBA:* rs773409311 [34]
Brazil	**SNPs** *PICALM:* rs3851179 [19]
Mexico	**SNPs** ALDH1A1: rs3764435 [22] APOE: (wild type allele) & rs429358 [27] SYT11: rs729022, rs822508 & rs34372695 MTHFR: rs1801133 [23, 31] LRRK2: rs1491942 [23] SNCA: rs356203, rs356219, rs356220, rs2736990, rs3857059 & rs7684318 [26, 35, 36] SNCA haplotypes: rs356220 + rs356203 + rs7684318 + rs2736990 [35] **INDELs** *NR4A2:* rs35479735 [32] *NR4A2:* haplotypes: rs35479735 + rs34884856 [32]
LARGE-PD (Brazil, Chile, Colombia, Peru & Uruguay)	**SNPs** H2BW1: rs525496 [20] SNCA: rs356182 [32] **CNVs** *PRKN/PARK2* + *SNCA* + *PLA2G6:* Combined CNV burden across the three loci [21]

For specific details about ORs, allele and genotype configuration refer to Table 1.

Most of the gathered investigations used a candidate gene approach with seventeen hypothesis-driven studies, and just two studies used a hypothesis-free methodology of genome-wide association study (GWAS) [32] and X-chromosome wide association study (XWAS) [20]. Different diagnostic criteria for idiopathic PD were reported, being the UK Parkinson’s Disease Society Brain Bank Diagnostic Criteria (UKPDSBB) [38] the most commonly used. The revised version of the Unified Parkinson’s disease rating scale [39] and a pure syndromic approach [27] supported idiopathic PD diagnosis as well. Among the main populations represented, the countries of Mexico, Brazil, Peru and Colombia stood out as the most studied regions, both in local studies and consortiums such as LARGE-PD. Of note, the growth of the LARGE-PD consortium patient cohort (Brazilian, Chilean, Peruvian, Colombian and Uruguayan subjects) is evident by the different number of patients each study included by increasing date of publication. In total, more than three-hundred genetic mutations across twenty-nine different loci were tested directly as genetic markers for idiopathic PD in Latin Americans. Out of these, twelve loci hosted multiple variants with statistically significant associations: seventeen SNPs, one INDEL and the combined burden of CNVs across three different loci.

SNP-type genetic variants showed a significant protective association against idiopathic PD in genes such as *PICALM* (rs3851179) in the Brazilian population, *H2BW1* (rs525496) in the LARGE-PD and replication cohort (XWAS), *ALDH1A1* (rs3764435) and *APOE-e3* in the Mexican population. On the other hand, SNPs in genes such as *LRRK2* (rs1491942), *MTHFR (*rs1801133), *SNCA (*: rs2736990, rs7684318, rs356203, rs356220, rs3857059, rs356219 and rs356182), *SYT11 (*rs822508, rs729022, rs34372695) and *APOE-e4* showed significant risk associations for idiopathic PD mainly in the Mexican population, in which haplotypes were found in the *SNCA* gene (across rs356220 + rs356203 + rs7684318 + rs2736990) with significant risk association. For the *GBA* gene, mutations classified as pathogenic for Gaucher disease were found to have a significant risk association with idiopathic PD, and particularly the rs773409311 variant had a significant risk association in the Colombian population, but its presence was not documented in their Peruvian cohort. Regarding INDEL-type variants, a significant risk association was found with the *NR4A2* variant (rs35479735**)**, for which haplotypes were identified that establish a risk association with idiopathic PD in the Mexican population. Finally, a significant risk association was identified when examining the burden of CNVs in genes typically associated with PD, mainly in *PRKN/PARK2*, *SNCA* and *PLA2G6* loci. No associations regarding VNTRs were found.

## Discussion

The findings gathered in this review disclose the genetic variants associated with idiopathic PD in the Latin America population, evidencing both risk and protective associations. Two high quality studies, Leal et al. [20] and Loesch et al. [32], used a hypothesis-free methodology identifying the rs525496 SNP near *H2BW1* as a protective variant and the rs356182 SNP in *SNCA* as a risk factor for idiopathic PD through XWAS and GWAS studies, respectively. On the other hand, seventeen studies employed a hypothesis-directed approach, testing more than three-hundred variants across twenty-nine different genetic loci and identifying fourteen SNPs associated with an increased risk (*GBA*: rs773409311; *SYT11*: rs822508, rs729022, rs34372695; *APOEε4*: rs42935358; *MTHFR*: rs1801133, *LRRK2*: rs1491942; *SNCA*: rs2736990, rs7684318, rs356203, rs356220, rs3857059, rs356219 and rs356182), three SNPs with a protective association (*PICALM*: rs3851179; *ALDH1A1*: rs3764435 and *APOE-ε3*), one INDEL at *NR4A*2 (rs35479735) and the cumulative burden of CNVs at *PRKN*, *SNCA* and *PLA2G6* as risk factors. In parallel, six SNP haplotypes in *SNCA* were found to be associated with an increased risk of the disease, while two INDEL haplotypes in *NR4A2* were linked to both risk and protective effects in idiopathic PD**.**

The identified genetic markers point to multiple neurodegenerative pathways relevant to idiopathic PD in Latin American populations, aligning with current hallmarks of neurodegeneration [40]. Accordingly, the products of the genes highlighted in this review are known to influence DNA and RNA defects (*H2BW1:* H2B histone family, member W*)* [41]*,* influence pathological protein aggregations *(SNCA:* alpha-synuclein and *GBA:* glucocerebrosidase*)* [42]*,* mediate synaptic and neuronal networks defects (*SYT11:* synaptotagmin 11 and *PRKN:* Parkin*)* [43]*,* promote inflammation *(APOE:* apolipoprotein E*, NR4A*: nuclear receptor subfamily 4 group A member 2 and *PLA2G6:* phospholipase A2 group VI*)* [44–46]*,* drive aberrant proteostasis (*GBA, PRKN*, *LRRK2:* Leucine-rich repeat kinase 2, *PICALM:* Phosphatidylinositol binding clatrhin assembly protein) [47–49]*,* and alter energy homeostasis (*MTHFR:* methylenetetrahydrofolate reductase and *ALDH1A:* aldehyde dehydrogenase 1*)* [50, 51]. Furthermore, this review outlines the overlap of pathophysiological pathways with other clinical entities, underscoring their shared genetic loci as monogenetic PD variants such as *PARK1*, *PARK2*, *PARK8* and *PARK14* share *SNCA*, *PRKN*, *LRRK2* and *PLA2G6* loci respectively with idiopathic PD (although not the exact variants) [52, 53]. Importantly, the mechanisms of monogenic and idiopathic PD are interconnected and not entirely distinct [54]. Moreover, genetic loci relevant to Alzheimer’s disease, such as *PICALM* and *APOE* [48], appear to converge with PD pathophysiological networks. This analysis underscores the potential of these genetic markers as future therapeutic targets.

Previous literature reviews on genetic markers for idiopathic PD in European populations [3, 7] have identified approximately 30 genetic susceptibility loci with more than 70 specific variants that influence the risk of PD, and most of them obtained through hypothesis-free studies [7]. In contrast, most of the genetic loci and mutations identified in this review came from hypothesis-directed studies, limiting validation of European findings. Nevertheless, Latin American efforts have managed to identify and overlap six genetic loci highly relevant in European population based on the results presented (*SYT11*, *GBA*, *SNCA*, *PRKN*, *LRRK2* and *PLA2G6*). Particularly, at validating specific European variants, Loesch et al. [32] found genome wide significance of rs356182 in *SNCA*, rs117615688 in *CRHR1*, rs1800547 and rs117615688 in *MAPT* loci in their LARGE-PD cohort; validating European GWAS reported by Nalls et al., 2019 [4]. Likewise, the SNP rs1491942 in *LRRK2* and rs356219 in *SNCA* of importance in Europeans [7] were reported by Romero-Gutierrez et al. [23] and Salas-Leal et al. [26] in Mexican cohorts. Also, the rs28602900 near *RPL10* previously identified in European population through XWAS analysis [55], was validated by Leal et al. [20] in the LARGE-PD discovery cohort. However, the variant missed significance within their Latin American replication cohort. On the other hand, speaking of novel genetic markers, the compiled evidence highlights *NR4A2, APOE, MTHFR, ALDH1A1, PICALM* and *H2BW1* as new complementary susceptibility loci for idiopathic PD in Latin Americans, extending and diversifying the genetic architecture of the disease. Relevant to novel loci, a variant near *NRROS* gene within LARGE-PD GWAS [32] was near statistically significant threshold, and further evidence will disclose if this new locus pertains to Hispanic idiopathic PD genetic architecture. Thus, there is a growing need to extend genomic research to classically underrepresented populations to enrich the genetic architecture of PD, not only for idiopathic PD but also familiar PD given the different deterministic mutations that drive the expression of monogenic PD in non-European regions compared with known European patterns [2].

Locally, many relevant genetic markers are yet to be replicated and validated across the diverse regions of Latin America. The variability in the genetic variants studied across the populations currently precludes the possibility of meta-analyzing the results on risk associations for the region as a whole or for specific subpopulations. However, promising findings have emerged; specific variants in *APOE, MTHFR* and *SNCA* [23, 26, 28, 31, 35] have been explored in few studies within the Mexican populations, yielding mixed outcomes except for the *MTHFR* rs1801133 variant which was statistically significant in all its studies [23, 31]. Additionally, the inherent population diversity in Latin America enriches the genetic landscape but introduces challenges in the study design. For instance, more than half of the LARGE-PD cohort comprises individuals of Peruvian origin and each region has different patterns of linkage disequilibrium [32]. Thus, important variants identified in Brazilian (e.g., *PICALM*) [19] and Colombian (e.g., *GBA*) [34] population by candidate gene analysis were not confirmed through GWAS analysis performed within LARGE-PD cohort, probablydue to the greater historical recombination and genetic diversity, since haplotypes within ancestries tend to differ in length (being shorter in African ancestry) [56]. Consequently, variants relevant in populations with higher African ancestry proportions, such as Colombia and Brazil (as reflected in the ancestry composition of the LARGE-PD sub-cohorts), may not be validated in cohorts from countries with a greater Native American ancestry component, such as Peru and Mexico [32, 57]. Similarly, variants specific to regions with lower European ancestry proportions (e.g., Peru) may not be detectable in populations with higher European ancestry proportions (e.g., Uruguay) [32]. Therefore, replication cohorts that closely match the global ancestry composition of the original study population are necessary to validate relevant genetic associations (see Table 2), along with methodologies that account for local ancestry within admixed populations [14]. Nonetheless, important efforts to overcome these limitations are highlighted, like the use of admixture mapping analysis. That has uncovered a locus on chromosome 14 (containing *STXBP6* gene), demonstrating importance in both the combined ancestry test and the Native American single-ancestry test [32]. Additionally, a locus on chromosome 6, containing the gene *RPS6KA2*, showed significance in the African single ancestry test [32]. Furthermore, a recent multi-ancestry meta-analysis by Kim et al., 2024 [6] revealed the importance of incorporating different ancestry in identification of novel risk loci for idiopathic PD; highlighting two independent loci that were close to strict GWAS significant threshold (JAK1 & H1SBP3) in Latin American and African sub-groups, that are pending to replicate in bigger cohorts. Finally, an emerging area of interest is the exploration of potential sex-dependent genetic effects in idiopathic PD. For instance, the GG genotype of *SNCA* rs3857059 has been associated with increased risk exclusively in females (OR: 1.31, 95% CI: 1.01–1.7) [36], while the protective effect of *ALDH1A1* rs3764435 remains significant only in males after sex-specific analysis [22]. Additionally, the protective locus rs525496 near *H2BW1* was also identified through an X-chromosome wide association study, highlighting sex differences in genomic susceptibility for the disease [20]. These findings collectively highlight the importance of incorporating sex-specific analyses in future research to validate these results and better understand their implications.

## Limitations & future directions

The discipline of PD genetics comes with unique challenges. In contrast to other complex neurodegenerative diseases such as Alzheimer’s, the penetrance of monogenic PD variants is incomplete and age-dependent, and can be influenced by polymorphisms [1] making it difficult to distinguish between Mendelian inheritance patterns and sporadic disease in selected cases. Fortunately, monogenetic cases are a minority and increasing access to next generation sequencing techniques could help to clarify doubts in these specific cases, along with exquisite clinical and family history taking. Also, the ever-changing clinical approach to idiopathic PD diagnosis, the different techniques to genotype the variants of interest and the heterogeneity of the populations coupled with the variability of candidate variant selection, limit the comparison of the results between studies and the performance of a meta-analysis. In addition, is worth to mention that novel and validated loci from European population summarized by this review in Latin American population remain to be further replicated in individual cohorts with matching ancestry patterns to underscore these discoveries. The findings of the present review highlight the need for increasing hypothesis-free studies within cohorts that preserve the proportion of Latin American diversity, and that considers admixture and sex-dependent effects in order confirm the current and future genetic markers. Finally, epistasis, functional genomics and therapeutic targets analysis along with genome-wide association studies with age at onset of idiopathic PD, its prognosis and pharmacogenomics of treatment response are future pathways that diverse genomic research should include in subsequent studies.

## Conclusions

The results of the present study contribute to the understanding of idiopathic PD genetics in Latin America and highlights potential genetic markers that aid disease in vivo diagnosis, identification of potential therapeutic targets, and strengthen the basis for prevention based on the knowledge of genetic susceptibility loci. Multiple genetic variants with significant risk and protective associations with idiopathic PD in the Latin American population, particularly SNPs, INDELs, CNVs, and haplotype clusters of SNPs and INDELs, were identified. Variants in genes such as *PICALM*, *H2BW1, ALDH1A1* and *APOE-ε3* showed a protective association, whereas variants in the *SNCA, NR4A2, PRKN, PLA2G6, GBA, APOE-ε4, MTFHR* and *LRRK2* genes exhibited a major risk association for idiopathic PD in populations from Mexico, Brazil, Colombia and LARGE-PD consortia cohort. There are, however, a limited number of studies addressing the genetics of PD in Latin America, and many of the genetic markers identified await replication and validation. This review reports genetic loci that aligns with existing European findings as well as novel loci that enhance the understanding of the genetic landscape of idiopathic PD. Hence, these findings highlight the genetic diversity in the Latin American population, and its contribution to the construction of the global genetic architecture of PD, while establishing the opportunity for the functional analysis of the genes identified, their possible relationship with environmental factors and new therapeutic targets. Further genetic studies enabling the development of personalized diagnostic and treatment strategies in idiopathic PD in Latin America are encouraged, due to the increasing prevalence of PD [58] and utility of genetic testing [59] in this region.

## Supplementary Information

Below is the link to the electronic supplementary material.

ESM 1 (PDF 46.4 KB)

## Data Availability

Availability of data and materials: full database search strategies and disclosed reasons of exclusion by full-text screening are available within supplementary material.
