# Supplementary material for: Genetic variants associated with idiopathic Parkinson’s disease in Latin America: A systematic review
Source: Neurogenetics. 2025 Apr 3;26(1):43. doi: 10.1007/s10048-025-00817-8 (PMC11968493; doi:10.1007/s10048-025-00817-8)
Supplement: Supplementary file 1 — (PDF 46.4 KB) [file 10048_2025_817_MOESM1_ESM.docx]

**Genetic variants associated with idiopathic Parkinson’s disease in Latin America:**
**A systematic review (Journal: Neurogenetics)**

Felipe Duarte-Zambrano^1, 2^, David Felipe Alfonso-Cedeño^1^, Jorge A. Barrero^1^, Luis Alejandro Rodríguez-Vanegas^1^, Valentina Moreno-Cárdenas^1^, Anamaría Olarte^1^, Gonzalo Arboleda^2, 3^, Humberto Arboleda^1, 2, 4^

^1^Semillero de investigación en Neurociencias, Facultad de Medicina, Universidad Nacional de Colombia, Bogotá, Colombia.

^2^Grupo de Investigación en Neurociencias y Muerte Celular, Facultad de Medicina e Instituto de Genética, Universidad Nacional de Colombia, Bogotá, Colombia

^3^Departamento de Patología, Facultad de Medicina, Universidad Nacional de Colombia, Bogotá, Colombia

^4^Departamento de Pediatría e Instituto de Genética, Facultad de Medicina, Universidad Nacional de Colombia, Bogotá, Colombia

Corresponding author: Felipe Duarte-Zambrano, email: jduartez@unal.edu.co

# **APPENDIX**

## APPENDIX 1: Search strategies.

| Parameter | Review report |
| --- | --- |
| Type of study | Systematic review |
| Database | MEDLINE |
| Platform | PubMed |
| Search date | December 14, 2023 |
| Time restriction | No time restriction |
| Language restriction | English and Spanish |
| Other restrictions | None |
| Search strategy | ("latin america*"[Title/Abstract] OR "latino*"[Title/Abstract] OR ("Antigua"[Title/Abstract] AND "Barbuda"[Title/Abstract]) OR "argentin*"[Title/Abstract] OR "Bahamas"[Title/Abstract] OR "Belize"[Title/Abstract] OR "bolivia*"[Title/Abstract] OR "brazil*"[Title/Abstract] OR "chile*"[Title/Abstract] OR "colombia*"[Title/Abstract] OR "costa rica"[Title/Abstract] OR "cuba*"[Title/Abstract] OR "dominica*"[Title/Abstract] OR "dominican republic"[Title/Abstract] OR "ecuador*"[Title/Abstract] OR "el salvador"[Title/Abstract] OR "Grenada"[Title/Abstract] OR "Guatemala"[Title/Abstract] OR "Guyana"[Title/Abstract] OR "Haiti"[Title/Abstract] OR "Honduras"[Title/Abstract] OR "Jamaica"[Title/Abstract] OR "mexic*"[Title/Abstract] OR "Nicaragua"[Title/Abstract] OR "Panama"[Title/Abstract] OR "Paraguay"[Title/Abstract] OR "peru*"[Title/Abstract] OR ("saint kitts"[Title/Abstract] AND "Nevis"[Title/Abstract]) OR "saint lucia"[Title/Abstract] OR ("saint vincent"[Title/Abstract] AND "the grenadines"[Title/Abstract]) OR "Suriname"[Title/Abstract] OR ("Trinidad"[Title/Abstract] AND "Tobago"[Title/Abstract]) OR "uruguay*"[Title/Abstract] OR "venezuela*"[Title/Abstract]) AND ("genetic loci"[Title/Abstract] OR "loci genetic"[Title/Abstract] OR "locus genetic"[Title/Abstract] OR "genetic locus"[Title/Abstract] OR "genotyp*"[Title/Abstract] OR "mutation*"[Title/Abstract] OR "genetic variation*"[Title/Abstract] OR "variations genetic"[Title/Abstract] OR "variation genetic"[Title/Abstract] OR "diversity genetic"[Title/Abstract] OR "diversities genetic"[Title/Abstract] OR "genetic diversities"[Title/Abstract] OR "genetic diversity"[Title/Abstract] OR "genetic variant*"[Title/Abstract] OR "polymorphism*"[Title/Abstract] OR "polymorphism genetic"[Title/Abstract] OR "polymorphisms genetic"[Title/Abstract] OR "genetic polymorphism"[Title/Abstract] OR "genetic polymorphisms"[Title/Abstract] OR "gene polymorphism"[Title/Abstract] OR "gene polymorphisms"[Title/Abstract] OR "polymorphism gene"[Title/Abstract] OR "polymorphisms gene"[Title/Abstract] OR (("polymorphic"[All Fields] OR "polymorphics"[All Fields] OR "polymorphism s"[All Fields] OR "polymorphism, genetic"[MeSH Terms] OR ("Polymorphism"[All Fields] AND "Genetic"[All Fields]) OR "genetic polymorphism"[All Fields] OR "Polymorphism"[All Fields] OR "Polymorphisms"[All Fields]) AND "Genetics"[Title/Abstract]) OR (("polymorphic"[All Fields] OR "polymorphics"[All Fields] OR "polymorphism s"[All Fields] OR "polymorphism, genetic"[MeSH Terms] OR ("Polymorphism"[All Fields] AND "Genetic"[All Fields]) OR "genetic polymorphism"[All Fields] OR "Polymorphism"[All Fields] OR "Polymorphisms"[All Fields]) AND "Genetics"[Title/Abstract]) OR "polymorphism single nucleotide"[Title/Abstract] OR "nucleotide polymorphism single"[Title/Abstract] OR "nucleotide polymorphisms single"[Title/Abstract] OR "polymorphisms single nucleotide"[Title/Abstract] OR "single nucleotide polymorphisms"[Title/Abstract] OR "SNPs"[Title/Abstract] OR "single nucleotide polymorphism"[Title/Abstract] OR "indel mutation"[Title/Abstract] OR "indel mutations"[Title/Abstract] OR "mutation indel"[Title/Abstract] OR "insertion deletion mutation"[Title/Abstract] OR "insertion deletion mutation"[Title/Abstract] OR "insertion deletion mutations"[Title/Abstract] OR "mutation insertion deletion"[Title/Abstract] OR "insertions deletions mutation"[Title/Abstract] OR "insertions deletions mutation"[Title/Abstract] OR "insertions deletions mutations"[Title/Abstract] OR "mutation insertions deletions"[Title/Abstract] OR "indels mutation"[Title/Abstract] OR "indels mutations"[Title/Abstract] OR "mutation indels"[Title/Abstract] OR "dna copy number variations"[Title/Abstract] OR "dna copy number variation"[Title/Abstract] OR "copy number variation dna"[Title/Abstract] OR "dna copy number variants"[Title/Abstract] OR "dna copy number variant"[Title/Abstract] OR "copy number variants dna"[Title/Abstract] OR "dna copy number polymorphisms"[Title/Abstract] OR (("DNA"[MeSH Terms] OR "DNA"[All Fields]) AND "copy number polymorphism"[Title/Abstract]) OR "copy number polymorphisms"[Title/Abstract] OR "copy number polymorphism"[Title/Abstract] OR "polymorphism copy number"[Title/Abstract] OR "polymorphisms copy number"[Title/Abstract] OR "dna copy number changes"[Title/Abstract] OR "dna copy number change"[Title/Abstract] OR "copy number changes dna"[Title/Abstract] OR "Haplotypes"[Title/Abstract] OR "Haplotype"[Title/Abstract] OR "haplogroup*"[Title/Abstract] OR "linkage disequilibrium"[Title/Abstract] OR "disequilibrium linkage"[Title/Abstract] OR (("Disequilibrium"[All Fields] OR "Disequilibriums"[All Fields]) AND "Linkage"[Title/Abstract]) OR "linkage disequilibriums"[Title/Abstract] OR "linkage disequilibrium region"[Title/Abstract] OR "VNTR"[Title/Abstract]) AND ("parkinson disease"[Title/Abstract] OR "idiopathic parkinson s disease"[Title/Abstract] OR "lewy body parkinson s disease"[Title/Abstract] OR "parkinson s disease idiopathic"[Title/Abstract] OR "parkinson s disease lewy body"[Title/Abstract] OR "parkinson disease idiopathic"[Title/Abstract] OR "parkinson s disease"[Title/Abstract] OR "idiopathic parkinson disease"[Title/Abstract] OR "lewy body parkinson disease"[Title/Abstract] OR "primary parkinsonism"[Title/Abstract] OR "parkinsonism primary"[Title/Abstract] OR "paralysis agitans"[Title/Abstract]) |
| Identified records | 114 |

| Parameter | Review report |
| --- | --- |
| Type of study | Systematic review |
| Database | Embase |
| Platform | Embase |
| Search date | December 14, 2023 |
| Time restriction | No time restriction |
| Language restriction | English and Spanish |
| Other restrictions | None |
| Search strategy | ('latin america':ti,ab OR 'hispanic'/exp OR 'antigua and barbuda':ti,ab OR argentina:ti,ab OR bahamas:ti,ab OR barbados:ti,ab OR belize:ti,ab OR bolivia:ti,ab OR brazil:ti,ab OR chile:ti,ab OR colombia:ti,ab OR 'costa rica':ti,ab OR cuba:ti,ab OR dominica:ti,ab OR 'dominican republic':ti,ab OR ecuador:ti,ab OR 'el salvador':ti,ab OR grenada:ti,ab OR guatemala:ti,ab OR guyana:ti,ab OR haiti:ti,ab OR honduras:ti,ab OR jamaica:ti,ab OR mexico:ti,ab OR nicaragua:ti,ab OR panama:ti,ab OR paraguay:ti,ab OR peru:ti,ab OR 'saint kitts and nevis':ti,ab OR 'saint lucia':ti,ab OR 'saint vincent and the grenadines':ti,ab OR suriname:ti,ab OR 'trinidad and tobago':ti,ab OR uruguay:ti,ab OR venezuela:ti,ab) AND ('gene locus'/exp OR 't complex genome region':ti,ab OR 't-complex genome region':ti,ab OR 'gene loci':ti,ab OR 'gene locus':ti,ab OR 'genetic loci':ti,ab OR 'genetic locus':ti,ab OR 'locus, gene':ti,ab OR 'genotype'/exp OR 'genotype':ti,ab OR 'mutation'/exp OR 'gene alteration':ti,ab OR 'genome mutation':ti,ab OR 'mutation':ti,ab OR 'genetic variation'/exp OR 'genetic variation':ti,ab OR 'genome structural variation':ti,ab OR 'genomic structural variation':ti,ab OR 'non additive genetic variation':ti,ab OR 'variation (genetics)':ti,ab OR 'variation, genetic':ti,ab OR 'genetic polymorphism'/exp OR 'genetic polymorphism':ti,ab OR 'polymorphism (genetics)':ti,ab OR 'polymorphism, genetic':ti,ab OR 'dna polymorphism'/exp OR 'dna polymorphism':ti,ab OR 'deoxyribonucleic acid polymorphism':ti,ab OR 'gene polymorphism':ti,ab OR 'polymorphism, dna':ti,ab OR 'single nucleotide polymorphism'/exp OR 'polymorphism, single nucleotide':ti,ab OR 'single nucleotide polymorphism':ti,ab OR 'single nucleotide variant':ti,ab OR 'single nucleotide variation':ti,ab OR 'indel mutation'/exp OR 'indel':ti,ab OR 'indel mutation':ti,ab OR 'indels':ti,ab OR 'insertion and deletion':ti,ab OR 'insertion deletion':ti,ab OR 'insertions and deletions':ti,ab OR 'insertions deletions':ti,ab OR 'copy number variation'/exp OR 'cnv (copy number variation)':ti,ab OR 'dna copy number variation':ti,ab OR 'dna copy number variations':ti,ab OR 'copy number variation':ti,ab OR 'copy number variations':ti,ab OR 'variable number of tandem repeat'/exp OR 'minisatellite repeats':ti,ab OR 'variable number of tandem repeat':ti,ab OR 'variable number of tandem repeats':ti,ab OR 'vntr':ti,ab OR 'haplotype'/exp OR 'analysis, haplotype':ti,ab OR 'haplotype':ti,ab OR 'haplotype analysis':ti,ab OR 'haplotypes':ti,ab OR 'haplogroup'/exp OR 'haplogroup':ti,ab OR 'gene linkage disequilibrium'/exp OR 'disequilibrium, genetic linkage':ti,ab OR 'gene linkage disequilibrium':ti,ab OR 'genetic linkage disequilibrium':ti,ab OR 'linkage disequilibrium':ti,ab) AND ('parkinson disease'/exp OR 'lewy bodies of parkinson disease':ti,ab OR 'lewy bodies of parkinson`s disease':ti,ab OR 'lewy bodies of parkinsons disease':ti,ab OR 'lewy body parkinson disease':ti,ab OR 'lewy body parkinson`s disease':ti,ab OR 'lewy body parkinsons disease':ti,ab OR 'parkinson disease':ti,ab OR 'parkinson`s disease':ti,ab OR 'parkinsons disease':ti,ab OR 'idiopathic parkinsonism':ti,ab OR 'paralysis agitans':ti,ab OR 'primary parkinsonism':ti,ab) |
| Identified records | 209 |

| Parameter | Review report |
| --- | --- |
| Type of study | Systematic review |
| Database | LILACS |
| Platform | *Biblioteca virtual en salud* |
| Search date | December 14, 2023 |
| Time restriction | No time restriction |
| Language restriction | English and Spanish |
| Other restrictions | None |
| Search strategy | ((América Latina) OR (Latinoamerica) OR (Antigua y Barbuda) OR (Argentina) OR (Bahamas) OR (Barbados) OR (Belice) OR (Bolivia) OR (Brasil) OR (Chile) OR (Colombia) OR (Costa Rica) OR (Cuba) OR (Dominica) OR (República Dominicana) OR (Ecuador) OR (El Salvador) OR (Granada) OR (Guatemala) OR (Guyana) OR (Haití) OR (Honduras) OR (Jamaica) OR (México) OR (Nicaragua) OR (Panamá) OR (Paraguay) OR (Perú) OR (San Cristóbal y Nieves) OR (San Vicente y las Grenadinas) OR (Suriname) OR (Trinidad y Tobago) OR (Uruguay) OR (Venezuela)) AND ((Sitios Genéticos) OR (loci genéticos) OR (loci génicos) OR (locus genético) OR (locus génico) OR (lugares genéticos) OR (sitio genético) OR (Mutación) OR (Genotipo ) OR (Genotipos ) OR (Variación Genética) OR (diversidad genética) OR (Polimorfismo Genético) OR (Polimorfismo de Nucleótido Simple) OR (Polimorfismo de Nucleótido único) OR (SNPs) OR (Mutación INDEL) OR (mutación inserción-deleción) OR (mutación por inserciones-deleciones) OR (Variaciones en el Número de Copia de ADN) OR (cambio en el número de copias de ADN) OR (cambios en el número de copias de ADN) OR (polimorfismo del número de copias) OR (polimorfismo del número de copias de ADN) OR (polimorfismos del número de copias) OR (polimorfismos del número de copias de ADN) OR (variación en el número de copias de ADN) OR (variante del número de copias de ADN) OR (variantes del número de copias de ADN) OR (Repeticiones de Minisatélite) OR (loci VNTR) OR (repeticiones minisatélites) OR (locus de VNTR) OR (minisatélite) OR (número variable de repeticiones en tándem) OR (región VNTR) OR (región de VNTR) OR (repetición de minisatélite) OR (repetición de minisatélites) OR (repetición en tándem de número variable) OR (repetición en tándem variable) OR (repetición variable en tándem) OR (secuencia VNTR) OR (secuencia de VNTR) OR (secuencias VNTR) OR (secuencias de VNTR7) OR (Haplotipos) OR (Haplotipo ) OR (Desequilibrio de Ligamiento) OR (desequilibrio de enlace) OR (desequilibrio de enlaces)) AND ((Enfermedad de Parkinson) OR (enfermedad de Parkinson con cuerpos de Lewy) OR (enfermedad de Parkinson idiopática) OR (parkinsonismo primario) OR (parálisis agitante)) |
| Identified records | 130 |

## APPENDIX 2: Excluded studies by full text screening

| **Author and year** | **Title** | **Reason of exclusion** |
| --- | --- | --- |
| Amara et al., 2019 | GBA mutations p.N370S and p.L444P are associated with Parkinson's disease in patients from Northern Brazil | Without measures of association |
| Benitez et al., 2010 | Exploration of genetic susceptibility factors for Parkinson’s disease in a South American sample | Without measures of association |
| Cagni et al., 2017 | Association of BDNF Val66MET Polymorphism With Parkinson’s Disease and Depression and Anxiety Symptoms | Family history of  Parkinson's disease |
| Camacho et al., 2012 | High frequency of Parkin exon rearrangements in Mexican-mestizo patients with early-onset Parkinson's disease | Without differential analysis of genotypes or mutations |
| Campelo et al., 2017 | Variants in SNCA Gene Are Associated with Parkinson’s Disease Risk and Cognitive Symptoms in a Brazilian Sample | Family history of  Parkinson's disease |
| Chien et al., 2014 | Frequency of the LRRK2 G2019S mutation in late-onset sporadic patients with Parkinson’s disease | Without measures of association |
| Clark et al., 2003 | The Saitohin ‘Q7R’ polymorphism and tau haplotype in multi-ethnic Alzheimer disease and Parkinson's disease cohorts | Family history of  Parkinson's disease |
| Cornejo-Oivas et al., 2017 | Variable frequency of LRRK2 variants in the Latin American research consortium on the genetics of Parkinson’s disease (LARGE-PD), a case of ancestry | Family history of  Parkinson's disease |
| Dávila-Ortiz de Montellano et al., 2011 | Determinación de la frecuencia del haplotipo H1/H2 de MAPT en muestras de población mestiza mexicana | Without measures of association |
| De Carvalho Aguiar et al., 2008 | Genetic and environmental findings in early-onset Parkinson's disease Brazilian patients | Without measures of association |
| De Carvalho Guimarães et al., 2012 | Glucocerebrosidase N370S and L444P mutations as risk factors for Parkinson's disease in Brazilian patients | Without measures of association |
| Dos Santos et al., 2010 | Mutational analysis of GIGYF2, ATP13A2 and GBA genes in Brazilian patients with early-onset Parkinson's disease | Without measures of association  Family history of  Parkinson's disease |
| Duque et al., 2015 | Analysis of the LRRK2 p.G2019S mutation in Colombian Parkinson’s Disease Patients | Without measures of association |
| García et al., 2014 | Low prevalence of most frequent pathogenic variants of six PARK genes in sporadic Parkinson’s disease | Without measures of association |
| Godeiro-Junior et al., 2009 | PINK1 mutations in a Brazilian cohort of early-onset Parkinson's disease patients | Without measures of association |
| González-del Rincón et al., 2013 | The L444P GBA mutation is associated with early-onset Parkinson's disease in Mexican Mestizos | Without measures of association |
| Leal et al., 2023 | Genome wide association studies using SNP1 reveals new associated genes with Parkinson Disease in a Latino Cohort | Without measures of association  Publication type (Poster) |
| Loesch et al., 2022 | Polygenic risk prediction and SNCA haplotype analysis in a Latino Parkinson's disease cohort | Without measures of association |
| Longo et al., 2015 | Alpha-synuclein A53T mutation is not frequent on a sample of Brazilian Parkinson’s disease patients | Without measures of association |
| Lopez et al., 2007 | Apolipoprotein E ε4 allele is associated with Parkinson disease risk in a Mexican Mestizo population | Family history of  Parkinson's disease |
| Martinez et al., 2010 | PARKIN-coding polymorphisms are not associated with Parkinson's disease in a population from northeastern Mexico | Without measures of association |
| Mata et al., 2011 | Lrrk2 p.Q1111H substitution and Parkinson’s disease in Latin America | Family history of  Parkinson's disease |
| McGuire et al., 2011 | Association of DRD2 and DRD3 polymorphisms with Parkinson's disease in a multiethnic consortium | Without differential analysis for Latino cohort. |
| Milla-Neyra et al., 2018 | Ausencia de la mutación A53T del gen SNCA en una muestra de pacientes con Enfermedad de Parkinson en el Perú | Without measures of association |
| Miltenberger-Miltenyi et L., 2023 | Genetic risk variants in New Yorkers of Puerto Rican and Dominican Republic heritage with Parkinson’s disease | Without differential measures of association  for Latin cohort. |
| Miranda-Morales et al., 2019 | H1/H2 MAPT haplotype and Parkinson’s disease in Mexican mestizo population | Family history of  Parkinson's disease |
| Monroy-Jaramillo et al., 2014 | Genetic mutations in early-onset Parkinson's disease Mexican patients: Molecular testing implications | Without measures of association |
| Perez-Pastene et al., 2007 | Lrrk2 mutations in South America: A study of Chilean Parkinson's disease | Without measures of association |
| Perez-Pastene et al., 2007 | Association of GST M1 null polymorphism with Parkinson's disease in a Chilean population with a strong Amerindian genetic component | Without measures of association |
| Pimentel et al., 2008 | A study of LRRK2 mutations and Parkinson's disease in Brazil | Family history of  Parkinson's disease  Without measures of association |
| Raggio et al., 2013 | LRRK2 mutations and Parkinson's disease in the Uruguayan population | Without measures of association  Publication type (Poster) |
| Ramirez et al., 2011 | Association of Parkinson disease to PARK16 in a Chilean sample | Family history of  Parkinson's disease |
| Sesar et al., 2012 | Validation study of genetic factors contributing to Parkinson's disease in Spanish and Latin American populations | Without measures of association  Publication type (Poster) |
| Spitz et al., 2008 | Association between Parkinson's disease and glucocerebrosidase mutations in Brazil | Without measures of association |
| Tipton et al., 2020 | Genetic characterization of Parkinson's disease patients in Ecuador and Colombia | Without measures of association |
| Tipton et al., 2020 | Prevalence of GBA p.K198E mutation in Colombian and Hispanic populations | Inadequate diagnostic criteria for idiopathic Parkinson's disease |
| Torrealba-Acosta et al., 2021 | Clinical and Genetic Analysis of Costa Rican Patients With Parkinson's Disease | Without measures of association |
| Yescas et al., 2010 | Low frequency of common LRRK2 mutations in Mexican patients with Parkinson's disease | Without measures of association |

## APPENDIX 3: Excluded studies after updated search (February 7th, 2025)

| **Author and year** | **Title** | **Reason of exclusion** |
| --- | --- | --- |
| Chaparro-Solano et al., 2025 | Critical evaluation of the current landscape of pharmacogenomics in Parkinson's disease - What is missing? A systematic review. | Secondary evidence (systematic review) |
| Cornejo-Olivas et al., 2025 | Genetic analysis of *APOE* reveals distinct origins and distribution of ancestry-enrichment haplotypes in the Mexican Biobank | Non-PD study |
| Koros et al., 2024 | A Global Perspective of GBA1-Related Parkinson's Disease: A Narrative Review. | Secondary evidence (narrative review) |
| Flroes-Ponce et al., 2024 | Establishment of induced pluripotent stem cell lines derived from Parkinson's disease Mexican patients: A sporadic (UNAMi002-A) and a familial (UNAMi003-A) case carrying a mutation in PINK1. | Family history of  Parkinson's disease |
| Oas et al., 2024 | Participant-reported personal utility of genetic testing for Parkinson’s disease and interest in clinical trial participation | Not a genetic association study |
| Lorenzo-Betancor et al., 2024 | Parkinson's Disease Gene Screening in Familial Cases from Central and South America | Family history of  Parkinson's disease |
| Jones et al., 2024 | Multi-ancestry population attributable risk assessment of common genetic variation in Alzheimer’s and Parkinson’s diseases | Combined analysis of AD and PD |
| Espindola et al., 2024 | Are genetic studies in Parkinson's disease necessary in clinical practice? Preliminary report of a cohort from Buenos Aires | Lacks control group |
| Ngo et al., 2024 | Lysosomal genes contribute to Parkinson’s disease near agriculture with high intensity pesticide use | Lacks control group |
| Quezada-Rivera et al., 2024 | Alteraciones genéticas asociadas a la enfermedad de Parkinson y Alzheimer: evolución y respuesta al tratamiento TT - Genetic Alterations Associated with Parkinson's and Alzheimer's Disease: Evolution and Response to Treatment | Secondary evidence (narrative review) |
| Fernandes et al., 2024 | A homozygous p.Val120Leu (c.358G > C) SOD1 mutation led to slowly progressive amyotrophic lateral sclerosis in a Brazilian family. | Non-PD study |
| Franklin et al., 2024 | The Huntington's Disease Gene Discovery. | Non-PD study |
| Flores-Lagunes et al., 2024 | First family with Perry syndrome from Mexico | Non-PD study |
| Gustavsson et al., 2024 | RAB32 Ser71Arg in autosomal dominant Parkinson's disease: linkage, association, and functional analyses | Family history of  Parkinson's disease |
| Cook et al., 2024 | Parkinson’s disease variant detection and disclosure: PD GENEration, a North American study | Lacks control group |
| Magalhães Pereira et al., 2024 | A systematic review and meta-analysis of the prevalence of Parkinson's disease in lower to upper-middle-income countries Check for updates | Secondary evidence (systematic review) |
| Kimball et al., 2024 | Genotype-Phenotype Spectrum of 52 Mexican Patients With Fabry Disease: A Novel GLA Variant With Atypical Phenotype. | Non-PD study |
| Benjamin et al., 2024 | Analysis of gene expression in the postmortem brain of neurotypical Black Americans reveals contributions of genetic ancestry | Post-mortem study |

## APPENDIX 4: Candidate genetic variants studied in Latin American population WITHOUT a statistically significant association with idiopathic PD (p<0.05).

| **SNPs** | | | | | |
| --- | --- | --- | --- | --- | --- |
| **Locus** | **Variant** | **PD association** | **Population** | **Author** | **Type of study/Quality** |
| ***NFKBIA*** | rs696 | No significant association | Brazil (55 cases vs 55 controls | Baltus et al., 2021 | Case-control /High |
| ***SNCA*** | rs2619364  rs2619363  rs17016074  rs356219    IVS4+66A-G      rs356219 | No significant association          No significant association  No significant association | Mexico: 171 cases vs 171 controls        Mexico: 51 cases vs 121 controls    Mexico: 118 cases vs 193 controls | Dávila-Ortiz de Montellano et al., 2016  Ramírez - Jirano et al. (2007)    Romero-Gutierrez et al., 2021 | Case-control /High          Case-control /Moderate    Case-control /High |
| ***RIC3*** | 293 variants with MAF > 1% | No significant association | LARGE PD: 798 cases vs 683 controls | Brolin et al, 2022 | Case-control /High |
| ***APOE*** | *ε2/ε3*  ε2/ ε2  ε4/ ε4  ε2/ε4  ε2 /ε2  ε2 /ε3  ε2 /ε4  ε3 /ε3  ε3 /ε4  ε4 /ε4 | No significant association        No significant association | Mexico: 105 cases vs 107 controls      Peru: 163 cases vs 176 controls | Gallegos-Arreola et al., 2009      Marca et al., 2013 | Case-control /Moderate        Case-control /Moderate |
| ***MT-ATP6*** | G8701A | No significant association | Mexico: 175 cases vs 194 controls | García et al. 2019 | Case-control /Low |
| **tRNAGln** | T4336C | No significant association | Mexico: 175 cases vs 194 controls | García et al. 2019 | Case-control /Low |
| ***USP24*** | rs13312 | No significant association | Mexico: 118 cases vs 193 controls | Romero-Gutierrez et al., 2021 | Case-control /High |
| ***PARK7*** | rs3766606 | No significant association | Mexico: 118 cases vs 193 controls | Romero-Gutierrez et al., 2021 | Case-control /High |
| ***NUCKS1*** | rs823128 | No significant association | Mexico: 118 cases vs 193 controls | Romero-Gutierrez et al., 2021 | Case-control /High |
| ***SLC41A1*** | rs823156 | No significant association | Mexico: 118 cases vs 193 controls | Romero-Gutierrez et al., 2021 | Case-control /High |
| ***GSK3B*** | rs334558 | No significant association | Mexico: 118 cases vs 193 controls | Romero-Gutierrez et al., 2021 | Case-control /High |
| ***DRD3*** | rs6280 | No significant association | Mexico: 118 cases vs 193 controls | Romero-Gutierrez et al., 2021 | Case-control /High |
| ***FAM47E/***  ***SCARB2*** | rs6812193 | No significant association | Mexico: 118 cases vs 193 controls | Romero-Gutierrez et al., 2021 | Case-control /High |
| ***PRKN (PARK2)*** | rs1801474  rs1801582 | No significant association | Mexico: 118 cases vs 193 controls | Romero-Gutierrez et al., 2021 | Case-control /High |
| ***ANKK1*** | rs1800497 | No significant association | Mexico: 118 cases vs 193 controls | Romero-Gutierrez et al., 2021 | Case-control /High |
| ***LRRK2*** | rs1994090 | No significant association | Mexico: 118 cases vs 193 controls | Romero-Gutierrez et al., 2021 | Case-control /High |
| ***MAPT*** | rs242562 | No significant association | Mexico: 118 cases vs 193 controls | Romero-Gutierrez et al., 2021 | Case-control /High |
| ***RAIL/***  ***SREBF1*** | rs11868035 | No significant association | Mexico: 118 cases vs 193 control | Romero-Gutierrez et al., 2021 | Case-control /High |
| ***CR1*** | rs3818361 | No significant association | Brazil: 166 cases vs 176 controls | Santos-Reboucas et al., 2017 | Case-control /High |
| ***CLU*** | rs11136000 | No significant association | Brazil: 166 cases vs 176 controls | Santos-Reboucas et al., 2017 | Case-control /High |
| ***SYT11*** | rs12563627 | No significant association | Mexico: 271 cases vs 260 controls | Sesar et al., 2016 | Case-control /High |
| ***GBA*** | rs439898  rs398123530  rs2230288  rs76763715  rs421016 | No significant association | Colombia: 131 cases vs 164 controls | Vélez-Pardo et al., 2019 | Case-control /Moderate |
| **INDELS** | | | | | |
| ***NFKB1*** | rs28362491 | No significant association | Brazil: 55 cases vs 55 controls | Baltus et al., 2021 | Case-control /High |
| ***NR4A2*** | rs34884856  (promoter) | No significant association | Mexico: 227 cases vs 454 controls | Ruiz-Sánchez et al., 2017 | Case-control /Moderate |
